# The Enhanced H_2_ Selectivity of SnO_2_ Gas Sensors with the Deposited SiO_2_ Filters on Surface of the Sensors

**DOI:** 10.3390/s19112478

**Published:** 2019-05-30

**Authors:** Xin Meng, Qinyi Zhang, Shunping Zhang, Ze He

**Affiliations:** 1 School of Materials Science and Engineering, Wuhan University of Technology, Wuhan 430070, China; Mengx1229@163.com (X.M.); He816414@163.com (Z.H.); 2 School of Materials Science and Engineering, Huazhong University of Science and Technology, Wuhan 430074, China; pszhang@mail.hust.edu.cn

**Keywords:** gas sensor, SiO_2_, hydrogen, selectivity, chemical vapor deposition

## Abstract

This paper reports a study on the enhanced H_2_ selectivity of SnO_2_ gas sensors with SiO_2_ on the surface of the sensors obtained via chemical vapor deposition using dirthoxydimethylsilane as the Si source. The gas sensors were tested for sensing performance towards ethanol, acetone, benzene, and hydrogen at operating temperatures from 150 °C to 400 °C. Our experimental results show that higher selectivity and responses to hydrogen were achieved by the deposition of SiO_2_ on the surface of the sensors. The sensor with SiO_2_ deposited on its surface at 500 °C for 8 h exhibited the highest response (R_a_/R_g_ = 144) to 1000 ppm hydrogen at 350 °C, and the sensor with SiO_2_ deposited on its surface at 600 °C for 4 h attained the maximum response variation coefficient (D = 69.4) to 1000 ppm hydrogen at 200 °C. The mechanism underlying the improvement in sensitivity and the higher responses to hydrogen in the sensors with SiO_2_ on their surface is also discussed.

## 1. Introduction

As an ideal clean energy source, hydrogen has widespread applications in the chemical industry, electronic field, aerospace industry, and civil engineering [1,2,3]. Given flammability and explosion of hydrogen, the safety and management of hydrogen energy present a stringent challenge. In order to solve this problem, the detection of hydrogen is required. The hydrogen gas sensor is one of the most effective unit to detect hydrogen [4,5].

A wide variety of hydrogen sensors have been developed, based on thermoelectric effects, catalytic burning (combustible gas sensors), metal oxide semiconductor (MOS), field effect transistor (FET), and surface acoustic wave (SAW) [6]. MOS gas sensors represent a class that have been extensively studied and successfully commercialized [7]. In terms of the recently published studies, MOS sensors exhibit excellent performance on humidity sensing [8,9,10]. Among the metal oxide semiconductor sensors, SnO_2_ sensors are widely used due to their low cost, high sensitivity, and good physical and chemical properties [11]. However, the lack of the anti-interference ability to other reducing gases limits their accuracy in the hydrogen detection process. Doping [12,13,14], filtering membranes [15,16,17,18], surface modification [19,20,21], and others are effective means to improve the selectivity of SnO_2_ gas sensors. In a related study, Lin et al. doped SnO_2_ with different concentrations of Ni. The result showed that the responses of the doped SnO_2_ sensors to the gases were two to eight times higher than that of the conventional SnO_2_ sensor [13]. In another related study, it is revealed by Fasaki et al. that SnO_2_ sensors modified by Au reduced the detection temperature of SnO_2_ to hydrogen from 180 °C to 85 °C, while increasing the response by approximately 50 times. Similar results can be achieved in SnO_2_ sensors modified by Pt [20]. 

Inspection of the published scientific literature indicates that one of the most efficient approaches to increase the selectivity of sensors is to use a filtering membrane [22], e.g., SnO_2_(Pd)/Al_2_O_3_(M) structure(M = Pt, Ru) [23], SnO_2_(Sb)/PdO_x_ nanocomposite [24], and more. Montmeat et al. observed that Pt film deposited on the surface of SnO_2_ by chemical vapor deposition (CVD) can effectively catalyze the oxidation of CO and C_2_H_5_OH at 500 °C [15]. Weber et al. developed highly efficient hydrogen sensors based on ZnO nanowires (NWs) coated with a thin layer of boron nitride (BN) decorated with palladium nanoparticles (NPs). Hydrogen gas could be detected for concentrations as low as 0.5 ppm [25]. In addition, since metal organic framework (MOF) materials e.g., ZIF-8, have a high specific surface area, they are often used as molecular sieves to improve the selectivity of the sensors [17,18,26,27]. For example, Matatagui et al. found that a combination of nanostructures of zeolitic imidazolate frameworks (ZIF-8 and ZIF-67) significantly improve the responses of the sensors as compared with that of ZIF-67 based sensors [17], which suggests that the adoption of the ZIFs membrane can enhance the selectivity of the gas sensors. Weber et al. confirmed the efficient use of the ZIF-8 nanomembrane to enhance the selectivity of ZnO NWs hydrogen sensors. Remarkably, high response signals were measured for H_2_ detection at low concentrations, whereas no noticeable response toward other tested gases, such as C_6_H_6_, C_7_H_8_, C_2_H_5_OH, and CH_3_COCH_3_, were detected [27]. In fact, the SiO_2_ membrane is one of the best filtering membranes to improve hydrogen selectivity of the SnO_2_ gas sensors and many excellent results have been reported [28,29,30,31]. Katsuki et al. prepared a SiO_2_ accumulated dense layer near the surface of the SnO_2_ gas sensors by CVD using hexamethyldisiloxane (HMDS) as the silicon source, which results in a prominent selectivity for H_2_ [28]. Wada et al. achieved similar results of selectivity for H_2_ [29]. Unlike the study by Katsuki et al. [28], triethoxymethylsilane (TEMS) and ethoxy-trimethylsilane (ETMS) were selected as the silicon source in the study by Wada et al. [29]. Hyodo et al. also reported that the variations in potential barrier height per grain boundary were increased and the H_2_ sensitivity of the SnO_2_ varistor-type sensors was improved when the SiO_2_ thin film was coated on surfaces of the sensors [30]. Tournier et al. presented a highly selective H_2_ sensor with minimum cross sensitivity to C_2_H_5_OH, CH_4_, and CO. After HMDS treatment at 600 °C for 6 h, the H_2_ sensitivity of the SnO_2_ thick film sensor with SiO_2_ deposited on its surface by CVD was increased to about 8.5 times that of the untreated SnO_2_ thick film sensor, whereas the sensitivities of the CVD treated sensor to C_2_H_5_OH, CH_4_, and CO were drastically reduced near 0 all over the temperature range [31]. Although the improvement in H_2_ selectivity of the sensors with the SiO_2_ filtering membrane has been reported in many published studies, the mechanism underlying the increased selectivity by adopting SiO_2_ membranes remains poorly understood. For example, it is hard to understand why the dense SiO_2_ layer could function as a molecular sieve [28], and it is unclear why the thickness of the SiO_2_ membrane is a key factor to uncover the mechanism of the selectivity, and to improve the performance of the sensors.

The present work investigates the effect of thickness of the SiO_2_ filtering membrane on sensitivities and selectivity of gas sensors, and the reasons responsible for selectivity improvement. In this paper, SiO_2_ was deposited on the SnO_2_ gas sensors by CVD using dirthoxydimethylsilane (DEMS) as the silicon source. The CVD-treated sensors can be fabricated using a simple and low-cost process. Thus, they have a good prospect of large-scale application. The testing results with hydrogen, ethanol, acetone, and benzene show that the selectivity and sensitivities of SnO_2_ gas sensors with SiO_2_ deposited on the surface to hydrogen have been significantly improved. The mechanism underlying the higher responses to hydrogen in the sensors with SiO_2_ on their surface was discussed.

## 2. Materials and Methods

### 2.1. Preparation of SnO_2_ Sensors

**Figure 1:**
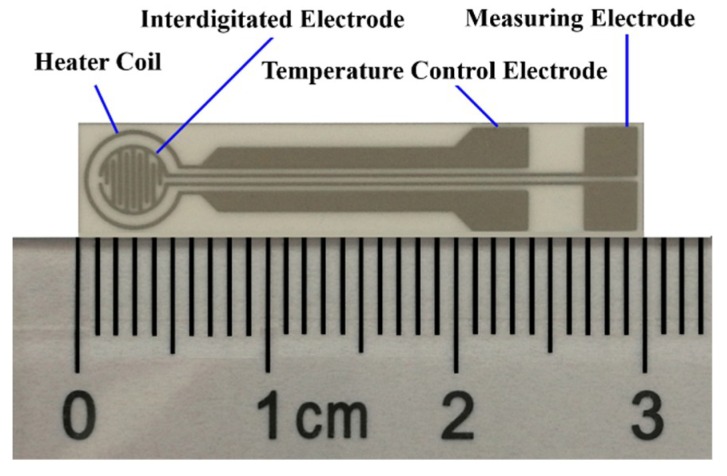
The TC-5010 sensor substrate.

Pastes consisting of commercial SnO_2_ powders and printing oil (YY-1010, Wuhan Huachuang Ruike Co., Ltd., Wuhan, China) with a mass ratio of 1:1 were deposited on the TC-5010 sensor substrates (Wuhan Huachuang Ruike Co., Ltd., Wuhan, China) by the screen-printing technology. The interdigitated Pt electrodes were printed on the substrate made of Al_2_O_3_ ceramic sheets by mechanically automated screen-printing technology, as shown in Figure 1. The printed sensors were dried at 60 °C for 1 h, and calcined at 600 °C for 2 h.

### 2.2. Chemical Vapor Deposition Treatment

**Figure 2:**
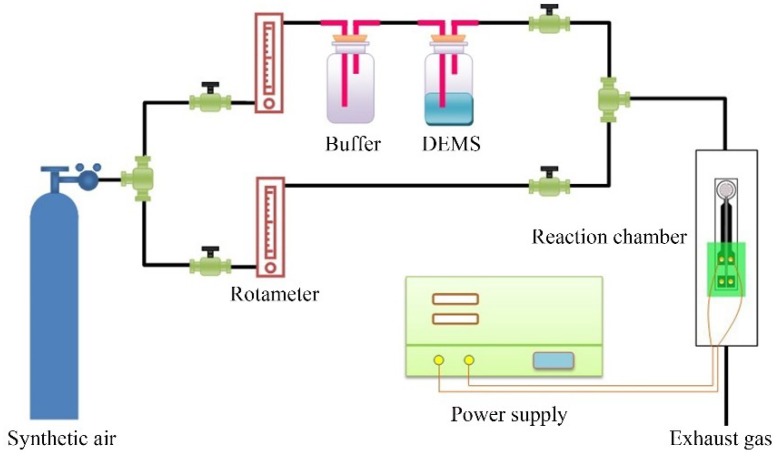
Schematic diagram of the chemical vapor deposition device.

Dirthoxydimethylsilane (DEMS) is chosen as the silicon source for the CVD treatment. The CVD processing apparatus is schematically shown in Figure 2. Before the CVD treatment, dry air was introduced into the reaction chamber at the flow rate of 50 ml/min for 10 min to dispel the gas therein. After the impurity gas was removed completely, the sensors were energized by the power supply. Because the electrical resistance of the Pt heater coil (Figure 1) is proportional to temperature, there is a proportional relationship between the power on the coil and the temperature of the sensors. The relationship between the powers and the temperatures was measured in advance and the used parameters of the power supply are shown in Table 1. After preheating, the valve of the dry air passage was closed, and the valve of the DEMS channel was opened simultaneously. The sensor substrate was treated in a DEMS atmosphere at the flow rate of 50 ml/min for 2 h, 4 h, 6 h, 8 h, and 10 h, respectively. 

The phases in the gas sensors were identified via X-ray diffraction analysis (XRD, D8 Advance, Bruker, Karlsruhe, Germany). The elements on the surface of the sensors were analyzed by an energy dispersive spectrometer (EDS, Zeiss Ultra Plus, Carl Zeiss AG, Jena, Germany). The surface morphology of the gas sensors was observed by a scanning electron microscope (SEM, Zeiss Ultra Plus, Carl Zeiss AG, Jena, Germany). The cross sections of the different sensors were observed by a scanning electron microscope (SEM, S-4800, HITACHI, Tokyo, Japan).

### 2.3. Measurement of Sensing Performance

**Figure 3:**
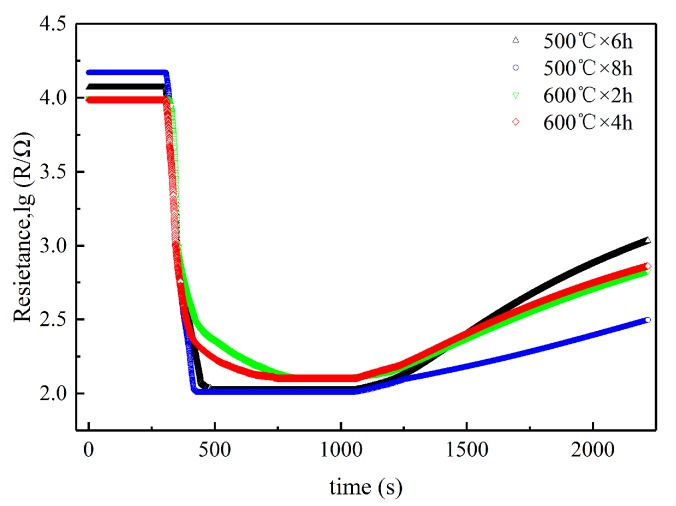
Response transients of the sensors to 1000 ppm H_2_ with synthetic air as carrier gas at 350 °C.

Gas-sensing performance was measured by a commercial SD-101 gas sensing performance testing device (Wuhan Huachuang Ruike Tech. Co. LTD, Wuhan, China), which can be simultaneously used to test four gas sensors. The gas sensors were tested under a voltage of 10 V at temperatures ranging from 150 °C to 450 °C attained by automatically adjusting the power supply of the heater coil by using a micro-processor. Volatile gases, including ethanol, acetone, and benzene, were measured by a static method at the concentration of 100 ppm (*v*/*v*), and H_2_ was tested by a dynamic method at the concentration of 1000 ppm (*v*/*v*). Dry synthetic air was used as a carrier gas in all tests, which consists of N_2_ and O_2_ at the volume ratio of 4:1. During the entire tests, the ambient temperature is 18 to 20 °C. The details on the test procedure for the gas sensing performance can refer to our previous work [32]. The synthetic air was first introduced into the testing chamber at a flow rate of 250 mL/min until the responses of the gas sensors was stabilized. Then, the mixture of 1000 ppm H_2_ in N_2_ with a flow rate of 200 mL/min and pure O_2_ with a flow rate of 50 mL/min, as the testing gas, was introduced into the chamber by adjusting a four-way valve. Figure 3 shows the response transients of the sensors to 1000 ppm H_2_ at 350 °C. It is clear that all the sensors exhibit stable responses both in air and in testing gas. In addition, the CVD-treated sensors responded quickly to hydrogen, but they took a long time to recover.

The response (S) of the sensor is defined as the ratio of the electrical resistance of the sensor in air (R_air_) to that in the test gas (R_gas_) (see Equation (1)). In order to compare the selectivity of the sensors, the response variation coefficient (D) is defined as the ratio of the response of any CVD-treated sensors (S_x_) to that of the untreated sensor (S_0_) under the same conditions (to the same testing gas, worked at the same temperature), as shown in Equation (2).

(1) $$S=\frac{R_{\mathrm{air}}}{R_{gas}}$$

(2) $$D=\frac{S_{x}}{S_{0}}$$

## 3. Results and Discussion

### 3.1. Characterization of the Gas Sensors

**Figure 4:**
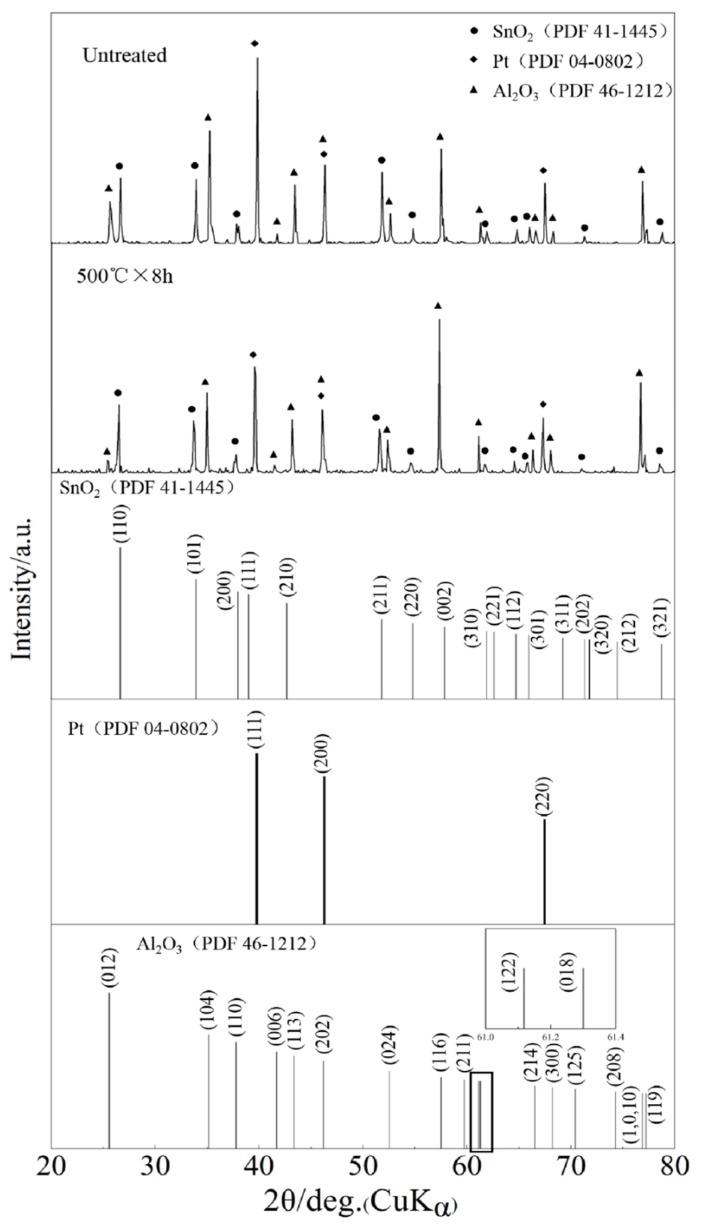
XRD patterns of the untreated and CVD-treated sensors, in comparison with peak positions in powder diffraction files of relevant phases.

Figure 4 shows the XRD pattern of the SnO_2_ sensor untreated and the SnO_2_ sensor CVD-treated at 500 °C for 8 h. Given similarity in the XRD patterns of all CVD-treated SnO_2_ sensors, only the XRD pattern of the SnO_2_ sensor CVD-treated at 500 °C for 8 h is provided herein. The untreated and the CVD-treated sensors exhibit similar peak positions and phases including SnO_2_, Pt, and Al_2_O_3_, and these peak positions are also consistent with those present in powder diffraction files of SnO_2_, Pt, and Al_2_O_3_. Noteworthy is the difference in relative intensities of the peaks between the CVD-treated and untreated gas sensors. This may be attributed to the presence of other phases as a result of SiO_2_ deposited on the surfaces of sensors. In order to verify the presence of SiO_2_, EDS analysis was conducted.

**Figure 5:**
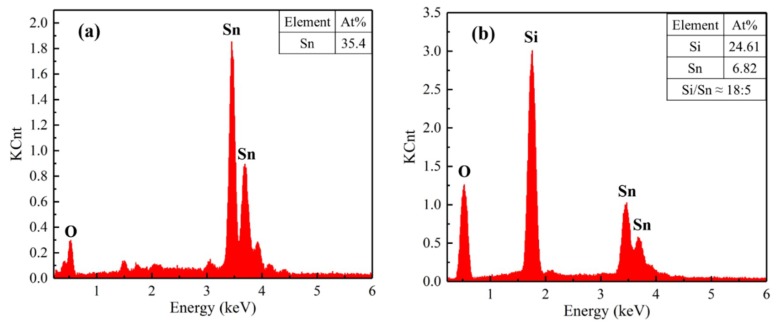
EDS spectrum of the sensors surfaces: (**a**) untreated and (**b**) CVD-treated at 600 °C for 4 h.

The EDS spectrum of the sensors’ surfaces are presented in Figure 5. On the one hand, as displayed in Figure 5a, the untreated SnO_2_ sensor surface contains only Sn and O elements. On the other hand, as shown in Figure 5b, the Si element can be detected on the surface of the CVD-treated sensors, which indicates the deposition of SiO_2_ on the surfaces of sensors. The absence of SiO_2_ peaks in the XRD pattern can be attributed to the small amount of the SiO_2_ deposited on surfaces of the sensors.

**Figure 6:**
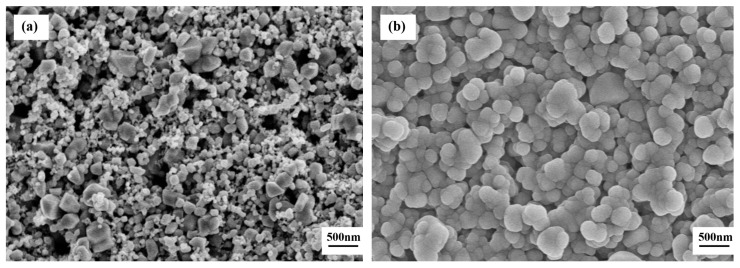

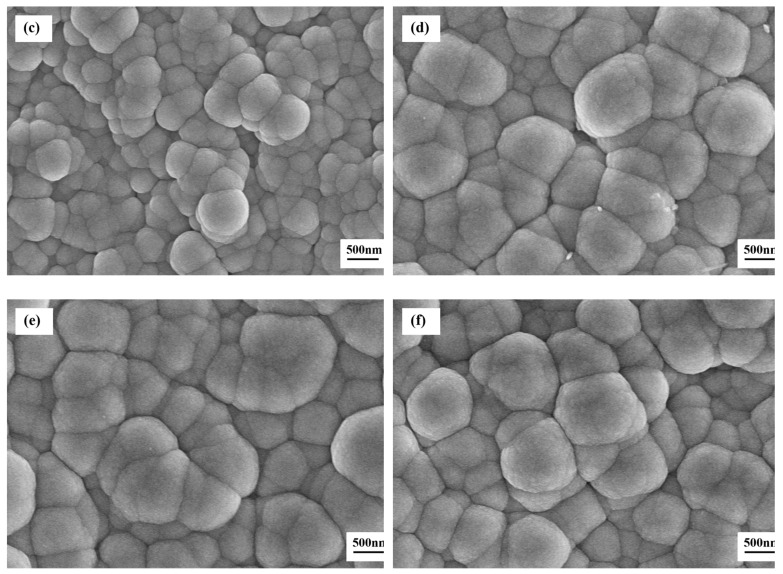
SEM micrographs of surface morphology of the sensors: (**a**) untreated, and CVD-treated at 500 °C for (**b**) 2 h, (**c**) 4 h, (**d**) 6 h, (**e**) 8 h, and (**f**) 10 h.

Figure 6 demonstrates the SEM micrographs of the sensors’ surfaces untreated and CVD-treated at 500 °C for a series of time periods. As shown in Figure 6a, spherical particles with the size of about 150 nm, polyhedral particles with the size of 200 to 400 nm, as well as many sintered macropores with the size of 200 to 400 nm can be observed. The sintered macropores were formed as a result of the volatilization of printing oil during calcination of the sensors. As shown in Figure 6b, in the CVD-treated sensors at 500 °C for 2 h, the SnO_2_ particles are coated by the spherical SiO_2_ particles with the size of about 250 nm, but some sintered macropores can still be seen despite being a smaller size than in the untreated sensors. With an increase in CVD treatment time to 4 h at 500 °C, the SnO_2_ particles were completely covered by the deposited SiO_2_ while the silica grew to about 450 nm in size, as displayed in Figure 6c. With a further increase in CVD treatment time to 6 h at 500 °C, the deposited SiO_2_ became denser but the SiO_2_ particle size coarsens to approximately 1μm, as shown in Figure 6d. As compared to the CVD-treated sensors at 500 °C for 6 h in Figure 6d, the surface morphology and the SiO_2_ particle sizes remain essentially unchanged in the sensors CVD-treated at 500 °C for 8 h and 10 h, as shown in Figure 6e,f.

**Figure 7:**
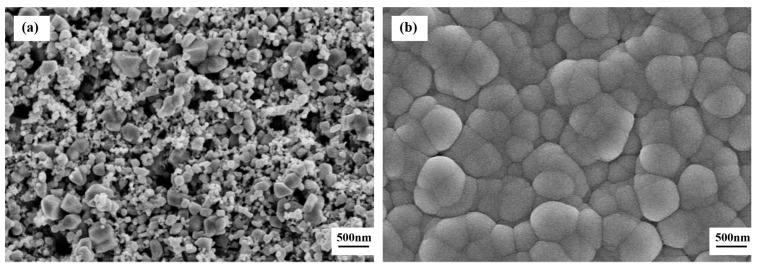

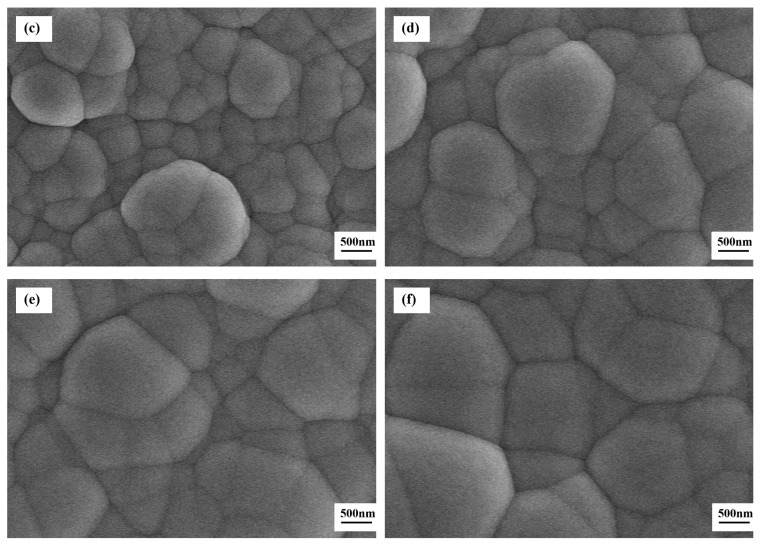
SEM micrographs of surface morphology of the sensors: (**a**) untreated and CVD-treated at 600 °C for (**b**) 2 h, (**c**) 4 h, (**d**) 6 h, (**e**) 8 h, and (**f**) 10 h.

Figure 7 shows the SEM micrographs of the surface morphology of the sensors untreated and CVD-treated at 600 °C for various time periods. The surface morphology of the CVD-treated sensors at 600 °C is similar to that of the CVD-treated sensors at 500 °C, given the same silicon source and processing method. The difference is that the SiO_2_ particle size of the CVD-treated sensors at 600 °C is larger due to the higher treatment temperature, as shown in Figure 7b–f. In the CVD-treated sensors at 600 °C, the SiO_2_ particle size increases from 500 nm with processing time of 2 h (Figure 7b) to 1.5 μm with processing time of 6–10 h (Figure 7d–f). The CVD treatments at 600 °C for 4 h and 500 °C for 8 h achieved the similar particle size of approximately 1 μm. In addition, the sintered macropores in sensors CVD-treated at 600 °C for 2 h have been completely covered by the SiO_2_ particles deposited on the surface. Based on the above discussion, the deposition rate of SiO_2_ to the substrate increases with an increase in CVD treatment temperature from 500 °C to 600 °C, as a result of the increased decomposition rate at a higher temperature.

**Figure 8:**
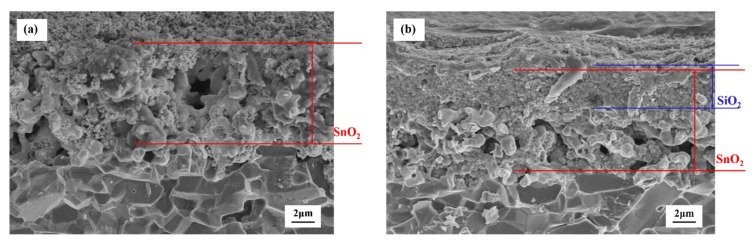
SEM micrographs of the cross-sections of the sensors: (**a**) untreated. (**b**) CVD treated at 500 °C for 8 h.

The SEM micrographs of the cross-sections of the sensors are shown in Figure 8. The cross-sectional morphology of the untreated sensor is characterized by the compact calcined SnO_2_ layer with the thickness of about 8.5 μm, as shown in Figure 8a. As shown in Figure 8b, the thickness of the CVD-treated sensor is almost the same as that of the untreated sensor. The CVD-treated sensor was more compact since the SiO_2_ particles are deposited between the SnO_2_ particles with the thickness of about 3.5 μm. The SiO_2_ not only covered the SnO_2_ particles but was also penetrated into the SnO_2_ layer through the sintered macropores between the SnO_2_ particles. Moreover, no stratification between the SnO_2_ layer and the SiO_2_ layer can be observed in the SEM micrographs of the sensor cross-section.

### 3.2. The Electrical Resistance of the Sensors in Air

**Figure 9:**
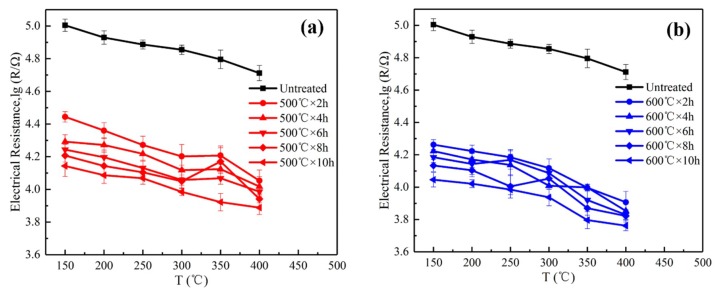
The electrical resistances of the sensors CVD treated at (**a**) 500 °C and (**b**) 600 °C as a function of temperature in air.

Figure 9 shows the electrical resistance of the sensors as a function of temperature in air, which indicates a tendency of the decrease of the electrical resistance with increasing temperature for each sensor. This can be ascribed to the decrement trend of SnO_2_ as a semiconductor material due to the increases of carriers at the condition of thermal excitation conditions [11].

In addition, Figure 9 reveals a significant reduction in the air electrical resistance of the CVD-treated sensors relative to those of the untreated sensors. The electrical resistance of the sensors at a constant temperature is critically affected by the amount of adsorbed O_2_ [32]. The absorbed O_2_ on the sensors seizes electrons from the SnO_2_ conduction band, which leads to a decrease in the number of carriers and, thus, higher electrical resistances of the sensors as a result of the thicker electron depletion layers. The decrease in the electrical resistances of the CVD-treated sensors in air originates from the presence of the compact SiO_2_ films that inhibit the O_2_ from entering the SnO_2_ by chemical adsorption. The thickness of the SiO_2_ deposited on the sensor increases with greater CVD treatment time, which results in a decrease in the amount of adsorbed O_2_ and, hence, the decrease of the electrical resistances of the sensors in air. Moreover, given that a higher treatment temperature generated more compact SiO_2_ films to inhibit the diffusion of oxygen, the CVD-treated sensors at 600 °C exhibited a lower electrical resistance in air than that of the CVD-treated sensors at 500 °C.

### 3.3. Sensing Responses to the Testing Gas

**Figure 10:**
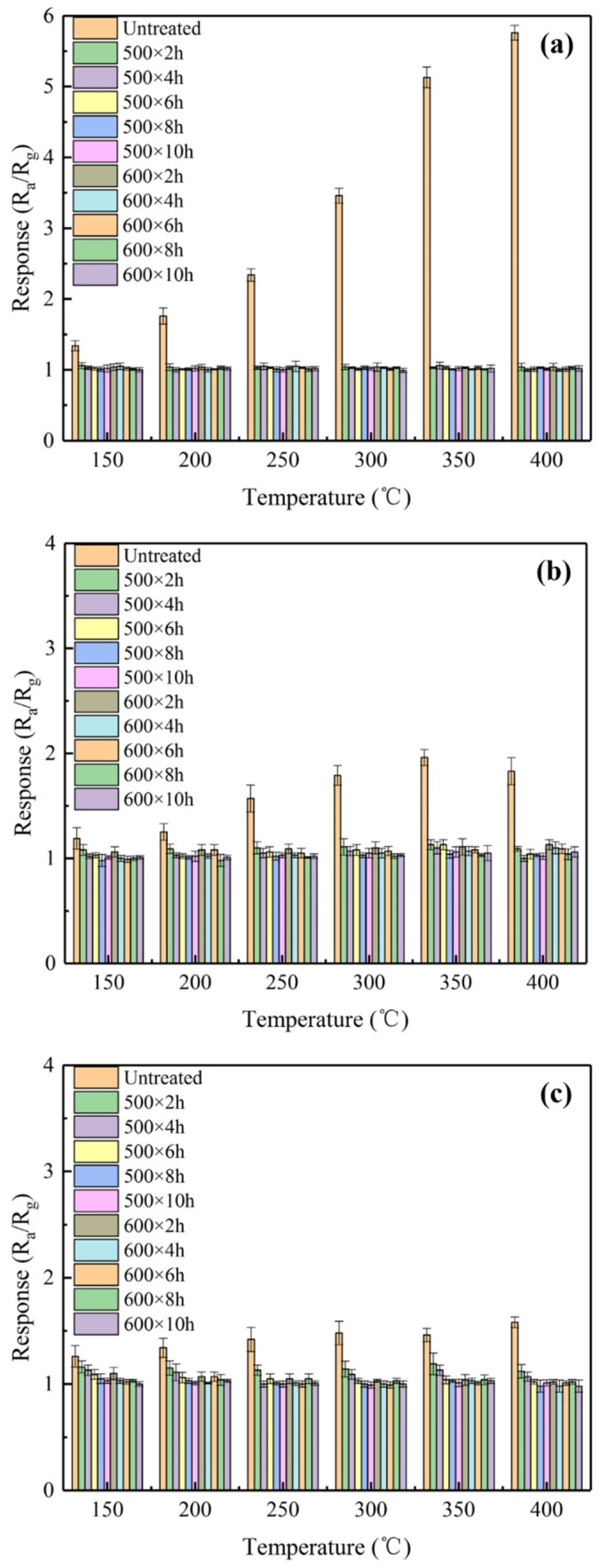
The responses of various sensors at a series of temperatures to (**a**) ethanol at 100 ppm, (**b**) acetone at 100 ppm, and (**c**) benzene at 100 ppm.

The responses of various sensors at a series of temperatures to ethanol, acetone, and benzene are demonstrated in Figure 10a–c, respectively. Inspection of Figure 10a gives rise to the following results. First, the responses of the untreated sensors to ethanol at 100 ppm increased slightly with an increase in the operating temperature up to 400 °C. Second, the responses of the CVD-treated sensors to 100 ppm ethanol were lower than those of the untreated sensor, due to the compact surfaces of the CVD-treated sensors. Third, the responses of the CVD-treated sensors to ethanol were so weak (R_a_/R_g_ ≈ 1) that they can be essentially neglected. Fourth, the response of the untreated sensors reached 5.76 to 100 ppm ethanol at 400 °C. Figure 10b,c present the following observations. First, the responses of the CVD-treated sensors to both acetone and benzene decreased significantly relative to those of untreated sensor. Second, the untreated sensors exhibited the largest responses to acetone (R_a_/R_g_ = 1.96) at 350 °C and to benzene (R_a_/R_g_ = 1.58) at 400 °C, respectively. Based on the above discussion, the optimum operating temperatures for untreated sensors fall in the range of 350 °C to 400 °C, consistent with the published studies [32]. 

**Figure 11:**
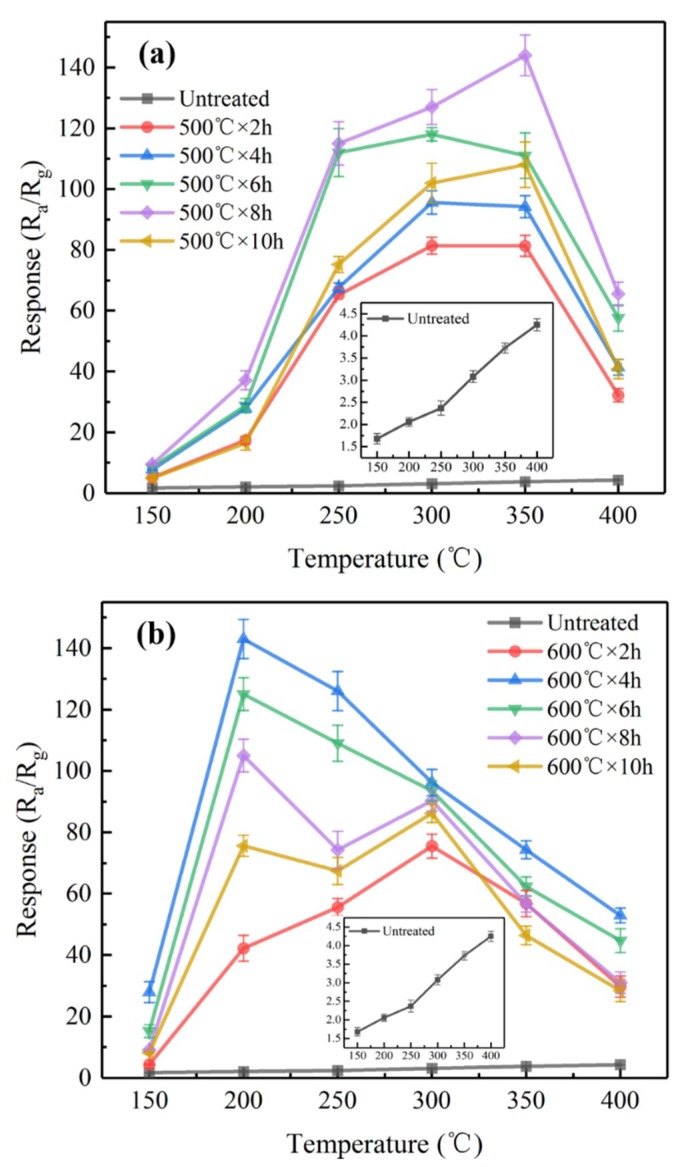
The responses of various sensors to 1000 ppm H_2_: (**a**) untreated and CVD treated at 500 °C, (**b**) untreated and CVD treated at 600 °C.

The responses of various sensors to 1000 ppm hydrogen as a function of temperatures are illustrated in Figure 11. The responses of the untreated sensors to hydrogen increased slightly when increasing the operating temperature up to 400 °C. At any temperatures, the responses of the CVD-treated sensors to hydrogen were significantly higher than those of the untreated sensors. The responses of the CVD-treated sensors to hydrogen exhibited a common tendency, i.e., first increased and then decreased with an increase in temperature. The optimum operating temperatures corresponding to the largest responses of the CVD-treated sensors were significantly reduced with an increase in the CVD treatment temperature. For example, the optimum operating temperatures of the CVD-treated sensors at 500 °C for 8 h and at 600 °C for 4 h are 350 °C and 200 °C, which correspond to the largest responses R_a_/R_g_ = 144 and R_a_/R_g_ = 143, respectively.

**Figure 12:**
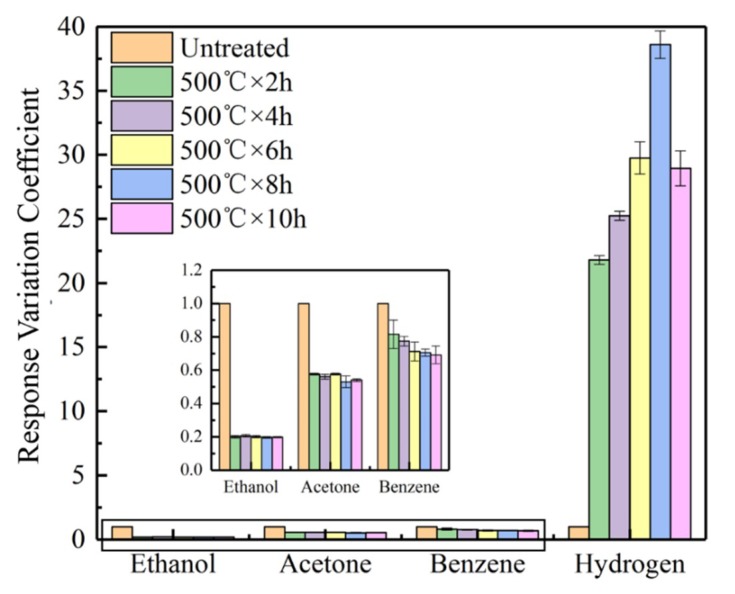
The response variation coefficients of the CVD-treated sensors at 500 °C with exposure to 1000 ppm hydrogen at 350 °C.

Figure 12 reports the response variation coefficients of the CVD-treated sensors at 500 °C when the sensors were exposed to 1000 ppm H_2_ at 350 °C. The response variation coefficients of the CVD-treated sensors at 500 °C to ethanol, acetone, and benzene was less than 1. In other words, the responses of the CVD-treated sensors to these gases were lower than those of the untreated sensors. This can be attributed to the SiO_2_ deposited on the sensor surfaces that prevented the detection to these gases by the SnO_2_ sensors. Figure 12 indicates that the sensors CVD-treated at 500 °C have a significantly high response variation coefficient to hydrogen, regardless of CVD treatment time. In particular, the sensors CVD-treated at 500 °C for 8 h exhibited the largest response variation coefficient (D = 38.6) to the hydrogen, i.e., the response of the sensor to H_2_ is increased by 38.6 times compared to that of the untreated sensor.

**Figure 13:**
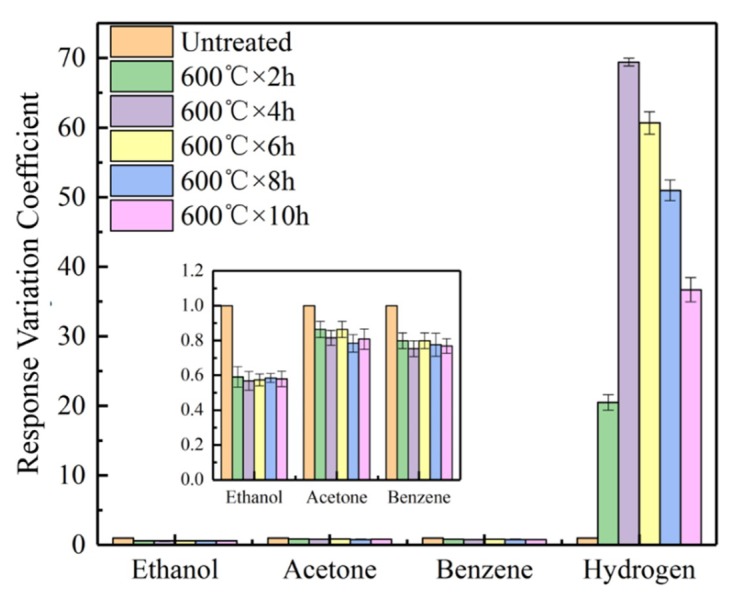
The response variation coefficients of the sensors CVD-treated at 600 °C with exposure to 1000 ppm hydrogen at 200 °C.

The response variation coefficient of the CVD-treated sensors at 600 °C with exposure to hydrogen at 200 °C is shown in Figure 13. Similar to the CVD-treated sensors at 500 °C, the sensors CVD-treated at 600 °C exhibited higher response variation coefficients to hydrogen and lower response variation coefficients to other gases, when compared to the untreated sensors. Noteworthy is that the sensors CVD-treated at 600 °C exhibited much higher response variation coefficients to hydrogen at 200 °C than those at 350 °C for the CVD-treated sensors at 500 °C. The maximum value (D = 69.4) of the response variation coefficient corresponds to CVD treatment at 600 °C for 4 h.

### 3.4. Discussion

**Figure 14:**
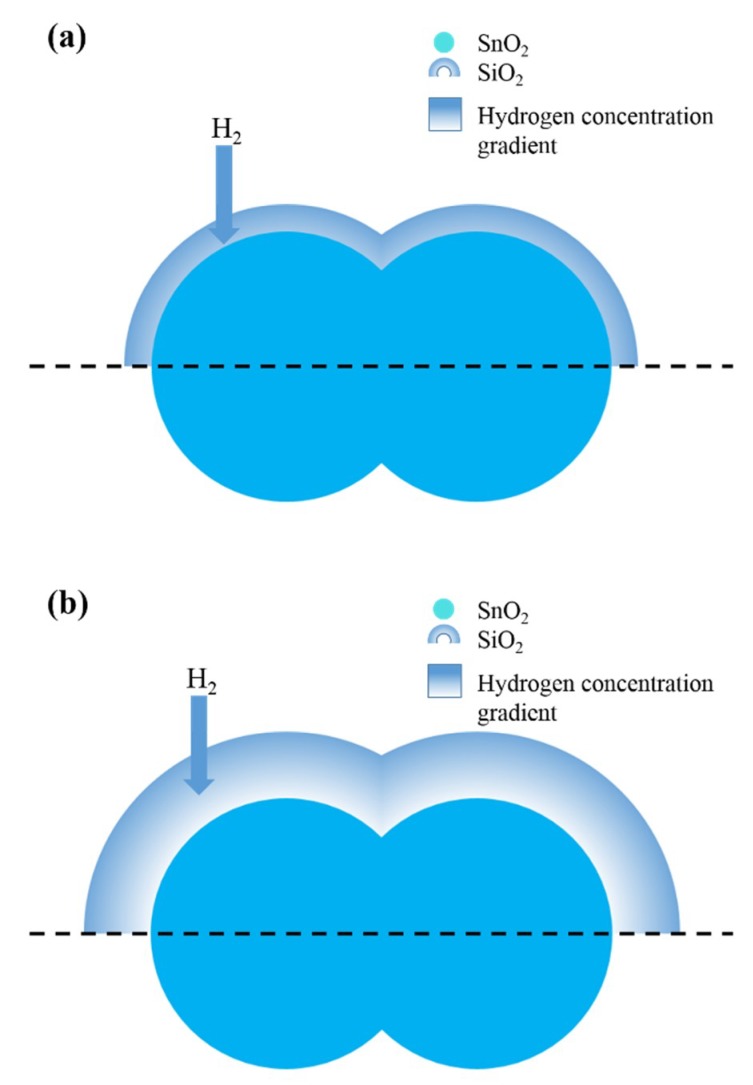
Schematic diagram showing the mechanism underlying gas sensing using SnO_2_ sensors with (**a**) thin SiO_2_ layer and (**b**) thick SiO_2_ layer deposited on their surfaces.

**Figure 15:**
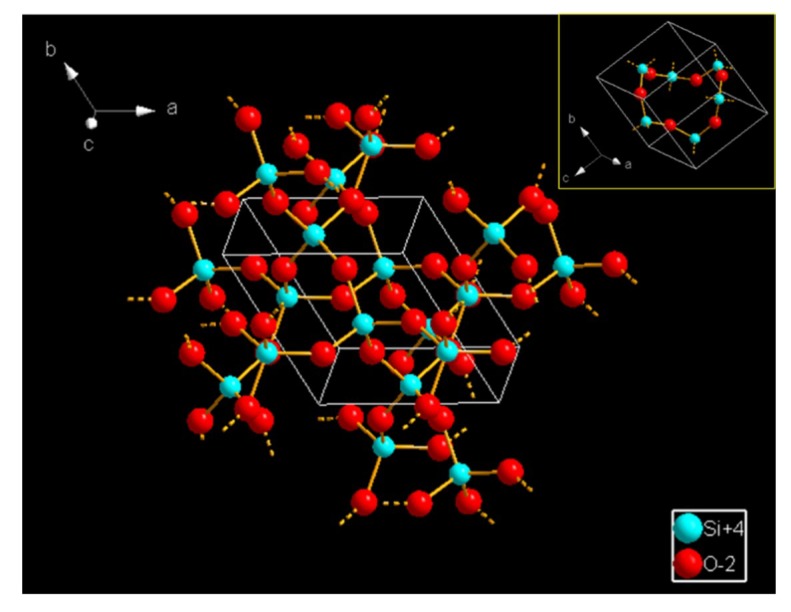
Schematic diagram of the crystal lattice of SiO_2._

Figure 14 schematically illustrates the mechanism underlying gas sensing using SnO_2_ gas sensors with SiO_2_ deposited on their surfaces, where the gas penetrates the SiO_2_ layer to reach SnO_2_ for detection. We suggest that the sizes of interstices inside the lattice of SiO_2_ deposited on the surfaces of SnO_2_ gas sensors critically influence the gas diffusion through SiO_2_, which results in selectivity of the gas sensors. Based on the crystal lattice of SiO_2_, as shown in the Figure 15, the size of interstices inside the lattice of SiO_2_ can be estimated as 3–4 Å. The interstices could be regarded as lattice pores between the atoms of silicon and oxygen. On the one hand, the kinetic diameter of H_2_ (2.89 Å) is on the same order of magnitude as that of the lattice pores inside SiO_2_. On the other hand, the sizes of molecules, including ethanol, acetone, and benzene, are much larger than the size of lattice pores inside SiO_2,_ and, consequently, the diffusion of these gases through the SiO_2_ layer to the sensing SnO_2_ layers is prohibited. Thus, the SiO_2_ layer deposited on the surfaces of SnO_2_ is responsible for excellent selectivity to hydrogen.

Given the hydrogen concentration inside the gas sensor is much lower than that in the atmosphere, the concentration gradient drives hydrogen to diffuse into the gas sensor. During diffusion, H_2_ becomes enriched in SiO_2_. H_2_ constantly penetrates the lattice pores of the SiO_2_ and accumulates in the SiO_2_, which leads to H_2_ enrichment in SiO_2_. As a result, the H_2_ concentration detected by the CVD-treated sensors is much higher than that in the atmosphere, which leads to the high responses of the sensors to hydrogen, as reported in Figure 14a. H_2_ enrichment increases with thickening of the SiO_2_ layers deposited by the CVD treatment. However, when the SiO_2_ layer reaches a critical thickness, the H_2_ concentration enriched in the deep layer of the SiO_2_ decreased. As a result, the H_2_ concentration detected by the sensors decreased, as shown in Figure 14b. This is responsible for the decreased response of the sensors to 1000 ppm hydrogen with increasing CVD treatment time.

Actually, improving the sensitivities and selectivity of hydrogen sensors has attracted considerable interest in recent years, as shown by the vast amount of the published studies [31,32,33,34,35,36]. The results in the present study and in the previously published studies are presented and compared in Table 2. In related studies, the mesoporous structure [32,33] and ZIFs [34,35] were frequently used as molecular sieves to improve the selectivity of the gas sensors. The preparations of mesoporous and ZIFs, however, are costly and complicated. Moreover, the sizes of the pores in mesoporous structures and ZIFs are on the order of nanometers and angstroms, respectively, which is much larger than the diameters of most gas molecules. The interstices in lattice, termed as lattice pores in the present study, have sizes comparable to the sizes of gas molecules. Consequently, the selectivity coefficients of the sensors significantly increased by depositing SiO_2_ on surfaces of gas sensors. Our experimental results suggest that the thickness of the SiO_2_ layers is critical to the performance of the sensors. The studies on the effect of SiO_2_ thickness for selecting the sensors progresses. 

## 4. Conclusions

The SnO_2_ sensor was modified by depositing SiO_2_ on the surface of the sensors using CVD with dirthoxydimethylsilane as the silicon source. Our experimental results show that the CVD-treated sensors exhibited excellent selectivity and sensitivities. The CVD-treated sensors have very high response values to hydrogen and low responses to ethanol, acetone, and benzene. In addition, the sensors CVD-treated at 500 °C for 8 h exhibited the highest response (R_a_/R_g_ = 144) to 1000 ppm hydrogen at 350 °C, and the sensors CVD-treated at 600 °C for 4 h had the maximum response variation coefficient (D = 69.4) to 1000 ppm hydrogen at 200 °C. The previously mentioned high selectivity and sensitivities can be attributed to the sieving effect on ethanol, acetone, and benzene molecules and the accumulation of hydrogen, both of which were induced by the deposited SiO_2_ layers. The future direction is to study the influence of humidity on long-term stability, response time, and recovery time of the CVD-treated sensors.

## Figures and Tables

**Table 1:** The temperatures of the sensors and the power supplied.

Temperature	Power
500 °C	3.9 W
600 °C	4.9 W

**Table 2:** Comparisons of the present study with the previously published studies.

Sensor System	H_2_ (ppm)	Interference Gases	Definition of the Response	Response (S_max_)	Response Variation Coefficient (D)	T (°C)
this work	1000	C_2_H_5_OH, C_6_H_6_, CH_3_COCH_3_	S = R_a_/R_g_	144	38.6	350
				143	69.4	200
SnO_2_-(m-SnO_2_) [32]	1000	C_2_H_5_OH, C_6_H_6_	S = R_a_/R_g_	22.2	~4.44	400
Pd-(ZIF-8) [34]	1000	O_2_, N_2_	S = ΔR/R_0_	0.3%	-	RT
ZnO-(ZIF-8) [35]	50	C_7_H_8_, C_6_H_6_	S = R_a_/R_g_	1.44	0.55	300
SnO_2_-SiO_2_ [31]	500	C_2_H_5_OH, CO	S = ΔR/R_0_	170	-	500
TiO_2_-Pd [36]	1000	C_2_H_5_OH, CO	S = ΔR/R_g_	139	-	180
m-SnO_2_ [33]	1000	-	S = R_a_/R_g_	43	2.69	350
